# From antiretroviral therapy access to provision of third line regimens: evidence of HIV Drug resistance mutations to first and second line regimens among Ugandan adults

**DOI:** 10.1186/s13104-016-2309-7

**Published:** 2016-12-23

**Authors:** Ivan Namakoola, Ivan Kasamba, Billy N. Mayanja, Patrick Kazooba, Joseph Lutaakome, Fred Lyagoba, Anne A. Kapaata, Pontiano Kaleebu, Paula Munderi, On behalf of the CoLTART study team

**Affiliations:** 1 MRC/UVRI Uganda Research Unit on AIDS, P.O. Box 49, Entebbe, Uganda; 2 MRC Tropical Epidemiology Group, London School of Hygiene and Tropical Medicine, Keppel Street, London, WC1E 7HT UK; 3 Department of Clinical Research, London School of Hygiene and Tropical Medicine, Keppel Street, London, WC1E 7HT UK

**Keywords:** Antiretroviral therapy, Drug resistance, Mutations, Third-line regimen

## Abstract

**Background:**

HIV care programs in resource-limited settings have hitherto concentrated on antiretroviral therapy (ART) access, but HIV drug resistance is emerging. In a cross-sectional study of HIV-positive adults on ART for ≥6 months enrolled into a prospective cohort in Uganda, plasma HIV RNA was measured and genotyped if ≥1000 copies/ml. Identified Drug resistance mutations (DRMs) were interpreted using the Stanford database, 2009 WHO list of DRMs and the IAS 2014 update on DRMs, and examined and tabulated by ART drug classes.

**Findings:**

Between July 2013 and August 2014, 953 individuals were enrolled, 119 (12.5%) had HIV-RNA ≥1000 copies/ml and 110 were successfully genotyped; 74 (67.3%) were on first-line and 36 (32.7%) on second-line ART regimens. The predominant HIV-1 subtypes were D (34.5%), A (33.6%) and Recombinant forms (21.8%). The commonest clinically significant major resistance mutations associated with the highest levels of reduced susceptibility or virological response to the relevant Nucleoside Reverse Transcriptase Inhibitor (NRTI) were; the Non-thymidine analogue mutations (Non-TAMS) M184V—20.7% and K65R—8.0%; and the TAMs M41L and K70R (both 8.0%). The major Non-NRTI (NNRTI) mutations were K103N—19.0%, G190A—7.0% and Y181C—6.0%. A relatively nonpolymorphic accessory mutation A98G—12.0% was also common. Seven of the 36 patients on second line ART had major Protease Inhibitor (PI) associated DRMS including; V82A—7.0%, I54V, M46I and L33I (all 5.0%). Also common were the accessory PI mutations L10I—27%, L10V—12.0% and L10F—5.0% that either reduce PI susceptibility or increase the replication of viruses containing PI-resistance mutations. Of the 7 patients with major PI DRMs, five had high level resistance to ritonavir boosted Lopinavir and Atazanavir, with Darunavir as the only susceptible PI tested.

**Conclusions:**

In resource-limited settings, HIV care programs that have previously concentrated on ART access, should now consider availing access to routine HIV viral load monitoring, targeted HIV drug resistance testing and availability of third-line ART regimens.

## Background

Antiretroviral therapy (ART) improves survival and quality of life of HIV-infected individuals and controls HIV transmission, however HIV-1 drug resistance limits the benefits of ART [1].

Globally, the number of HIV-positive people is increasing and the numbers of new HIV infections and mortality due to AIDS has declined. In 2014, globally 14.9 million (40%) people living with HIV were receiving ART, of which 13.5 million were in low- and middle-income countries [2]. In Uganda, 750,896 (50%) HIV infected people were receiving ART by December 2014 [3].

Despite the progress in the rapid ART scale up, HIV drug resistance profiling prior to starting ART, and routine virological monitoring and drug resistance testing are not yet standard HIV care in resource limited settings as is the case in the developed world [4]. The emergence of HIV drug resistance may limit the sustained benefits of ART in settings with limited laboratory monitoring and drug options. Drug resistance prevalence varies widely, at 2.8% in sub-Saharan Africa compared to 11.5% in North America, while in South Africa rising levels of acquired antiretroviral drug resistance and newly infected patients with resistant viruses have been reported [5, 6].

Although HIV care programs in resource limited settings have hitherto concentrated on expanding ART access, the emergency of HIV drug resistance is a challenge. We document the antiretroviral (ARV) drug resistance mutations and susceptibility patterns among Ugandan adults on ART. We also highlight the need for HIV care programs in resource limited settings to avail access to routine virological monitoring, access to targeted HIV drug resistance testing and alternative third line ART regimen drugs.

This was a cross-sectional study of HIV-positive adults (18 years and above) at enrolment into a prospective clinical cohort established to study the complications of long-term ART (CoLTART). Individuals on ART for 6 months or more were recruited from two ART cohorts; the Entebbe site of the former Development of Antiretroviral Therapy in Africa (DART) Trial cohort established in 2003 [7], and the former Rural Clinical Cohort in south western Uganda where ART was introduced in 2004 [8]. ART history was obtained from the former ART cohorts and included; dates of ART initiation and switches, ART regimens, baseline viral load and CD4 cell counts. First-line ART regimens consisted of two nucleoside reverse transcriptase inhibitors (NRTI) and one non-nucleoside reverse transcriptase inhibitor (NNRTI). Some participants in the DART Trial initiated ART with a triple nucleoside (3 NRTIs) first-line ART regimen. Second-line ART regimens consisted of one or two NRTIs and a boosted protease inhibitor (PI). Individual ARVs in an ART regimen were substituted in the event of adverse drug effects, contraindications by medical conditions or interactions with other concurrently administered medications while complete ART switches were made in the event of treatment failure.

CD4 cell counts were measured with the FACSCount or FACSCalibur machine (Becton–Dickinson, USA). Plasma HIV-1 RNA was quantified using the Cobas Ampliprep/Cobas Taqman 48 version 2.0 HIV-1 viral load assay, [Roche Molecular Diagnostics (RMD), NJ, USA]. Samples with HIV RNA viral load of 1000 copies/mL or higher were genotyped. For ARV drug resistance testing, HIV RNA was extracted using the QIAmp Viral RNA mini kit (Qiagen), the entire protease and amino terminus of the reverse transcriptase was amplified, cleaned and sequenced using the ABI 3500 machine (Applied Biosystems). Sequences were base-called using Sequencher v5.2.4 and sequence alignments performed using BioEdit v7.2.5 [9] and SeaView v4.0 [10]. HIV-1 subtyping was done using SCUEAL and REGA online software [11], and Recombinant Identification Program (RIP). Drug resistance mutations (DRMs) were interpreted using the Stanford HIVdb Program. Assigned DRMs were interpreted using the 2009 WHO list for epidemiological surveillance of TDR alongside the lAS 2014 Update of DRMs of HIV-1. Basic phylogenies were performed to determine sequence relatedness and to rule out contaminations. Sequences with genetic mixtures of wild-type and mutant sequences at amino acid sites that code for SDRMs were considered to be drug-resistant. Quality Assurance was done using the Calibrated Population Resistance tool (CPR), Stanford and the Los Alamos National database (LANL dbase) for the HIV Sequence Quality Analysis [12]. The Laboratory is WHO HIVDR Region accredited.

Participants with HIV-1 RNA viral load of 1000 copies/mL or higher, whose samples were successfully amplified and genotyped were included in this analysis. The socio-demographic characteristics including age and sex were examined and tabulated. ART data consisted of the type of ART regimen and duration on ART. The three ART regimen types were; Triple nucleoside (3 NRTIs), 2 NRTIs with a NNRTI, and a PI-based regimen. Duration on ART was grouped into below 5 years, 5–9 years, and above 9 years. Drug resistance mutations to one antiretroviral class only (either NRTI, NNRTI or PI), two classes only (either NRTI and NNRTI, PI and NRTI, or PI and NNRTI) or three antiretroviral classes (NRTI and 1 NNRTI and PI) were tabulated. For participants with a major PI DRM, we showed the resistance levels to each antiretroviral drug.

## Findings

Out of the 953 individuals on ART for 6 or more months, 119 (12.5%) had a viral load ≥1000 copies/ml. The five samples that did not amplify and the 4 with insufficient quantities were excluded from this analysis. Of the 110 individuals whose samples were sequenced for drug resistance testing, 73 (66.4%) were females, 95 (86.4%) were aged 35 years and above, 89 (80.9%) had been on ART for 9 or more years, with 74 (67.3%) on first and 36 (32.7%) on second-line ART regimens at enrolment. Sixty-five (59.1%) had CD4 cell counts of 350 cells/ml or less and 67 (60.9%) had HIV RNA of 10,000 copies/ml or more. The predominant HIV-1 subtypes were D (34.5%), A (33.6%) and Recombinant forms (21.8%) (Table 1). Of the 110 individuals with drug resistance testing results, 8 (7.3%) had no detectable drug resistance mutations (DRMs) while 102 (92.7%) had at least one detectable DRMs, distributed as follows: DRMs to any NRTI—92 (83.6%), to any NNRTIs—77 (70.0%) and to any PIs—33 (30.0%). DRMs by ART regimen line were: triple nucleoside—39 (35.5%), 2NRTI with an NNRTI—31 (28.2%) and second-line PI based regimen—33 (30.0%). Twenty-two participants (20.0%) had DRMs to one ARV class only (NRTI—14, NNRTI—7 and PI—1), 60 (54.5%) participants had dual-class DRMs (NRTI + NNRTI—48, PI + NRTI—10 and PI + NNRTI—2, while 20 (18.2%) had triple class DRMs (Table 2).

**Table 1:** Characteristics of participants with HIV viral loads ≥1000 copies/ml at enrolment by ART regimen line

Characteristic	All participants n (%)	ART regimen line	
		First line ART n (%)	Second line ART n (%)
All (n, row %)	110	74 (67.3)	36 (32.7)
*Study site*			
Entebbe	90 (81.8)	65 (87.8)	25 (69.4)
Kyamulibwa	20 (18.2)	9 (12.2)	11 (30.6)
*Sex*			
Females	73 (66.4)	53 (71.6)	20 (55.6)
Males	37 (33.6)	21 (28.4)	16 (44.4)
*Age, years*			
18–34	15 (13.6)	6 (8.1)	9 (25.0)
35–49	72 (65.5)	50 (67.6)	22 (61.1)
50+	23 (20.9)	18 (24.3)	5 (13.9)
*Body Mass Index (kg/m* ^*2*^ *)* ^a^			
<18.5	15 (13.6)	7 (9.5)	8 (22.2)
18.5–24.9	63 (57.3)	44 (59.5)	19 (52.8)
25.0–29.9	20 (18.2)	14 (18.9)	6 (16.7)
≥30	11 (10.0)	8 (10.8)	3 (8.3)
*HIV subtype*			
A	37 (33.6)	25 (33.8)	12 (33.3)
B	5 (4.5)	3 (4.1)	2 (5.6)
C	6 (5.5)	5 (6.8)	1 (2.8)
D	38 (34.5)	22 (29.7)	16 (44.4)
CRF01_AE	24 (21.8)	19 (25.7)	5 (13.9)
*CD4 cell counts at enrolment*—*cells/ml* ^b^			
≤350	65 (59.1)	41 (55.4)	24 (66.7)
351–500	26 (23.6)	22 (29.7)	4 (11.1)
501+	11 (10.0)	5 (6.8)	6 (16.7)
*Total duration on ART, years*			
0–<5	15 (13.6)	9 (12.2)	6 (16.7)
5–<9	6 (5.5)	2 (2.7)	4 (11.1)
9+	89 (80.9)	63 (85.1)	26 (72.2)
*Duration on current ART regimen, years* ^c^			
<5	17 (15.5)	9 (12.2)	8 (22.2)
5–<9	17 (15.5)	2 (2.7)	15 (41.7)
9+	63 (57.3)	63 (85.1)	0 (0.0)
*Viral loads (copies/ml)*			
1000–9999	43 (39.1)	29 (39.2)	14 (38.9)
10,000+	67 (60.9)	45 (60.8)	22 (61.1)

Missing variable data: a = 1 patient on first line ART, b = 8 patients (6 on first and 2 on second line ART), c = 13 patients on second line ART

**Table 2:** Drug resistance mutations (DRMs) of participants tested for drug resistance among those with HIV viral loads ≥1000 copies/ml at enrolment, by ARV class

Drug resistance mutation(s)	Proportion with amplified drug resistance tests, n (%)	Proportion with major PI mutations, n (%)^1^	Most common mutations^2^
*All*	110	7 (6.4)	
*No mutation detected*	8 (7.3)		
*At least one detected (NRTI, NNRTI, PI)*	102 (92.7)	7 (6.9)	
Any NRTI	92 (83.6)	6 (6.5)	*Non*-*TAMs:* M184V (20.7%), K65R (8.0%) *TAMs:* M41L (8.0%), K70R (8.0%)
Any NNRTI	77 (70.0)	4 (5.2)	K103N (19.0%), G190A (7.0%), Y181C (6.0%)
Any PI^3^	33 (30.0)	7 (21.2)	*Major:* V82A (7.0%) and I54V, M46I, L33I (all 5.0%) *Accessory:* L10I (27.0%), L10V (12.0%), L10F(5.0%)
*Mutation to 1 ARV class only (n* *=* *22)*			
NRTI	14 (12.7)	0 (0.0)	
NNRTI	7 (6.4)	0 (0.0)	
PI	1 (0.9)	0 (0.0)	
*Mutation to 2 ARV classes only (n* *=* *60)*			
NRTI + NNRTI	48 (43.6)	0 (0.0)	
PI + NRTI	10 (9.1)	3 (30.0)	
PI + NNRTI	2 (1.8)	1 (50.0)	
*Mutations to 3 ARV classes (n* *=* *20)* ^4^	20 (18.2)	3 (15.0)	
HIV subtype			
A	37 (33.6)	3 (8.1)	V82A/S, I54V/U, M46I/L, K43T, L10F, L89V, L24IL
B	5 (4.5)	0 (0.0)	
C	6 (5.5)	0 (0.0)	
D	38 (34.5)	4 (10.5)	V82A/V/F, I54IV, I54V, M46I, L33F, L10I, L10F, L24I, K20T, A71T, Q58E, I47V
CRF01_AE	24 (21.8)	0 (0.0)	

*TAMs* thymidine analogue mutations

^1^These are row percentages of participants with major PI mutations in each category with amplified drug resistance tests

^2^Denominator is all mutations to NRTI (n = 352), NNRTI (n = 144) or PI (n = 59)

^3^PI DRMs include 7 with a major and 26 with only minor PI DRMs

^4^Only major PI mutations are shown for HIV subtypes

In our cohort, the commonest clinically significant major NRTI resistance mutations associated with highest levels of reduced susceptibility or virological response were the Non-thymidine analogue mutations (Non-TAMs): M184V—20.7% and K65R—8.0%, while M41L and K70R (both 8.0%) were the commonest TAMs. The commonest major NNRTI resistance mutations known to reduce susceptibility or virological response to NNRTIs were: K103N—19.0%, G190A—7.0% and Y181C—6.0%. A relatively nonpolymorphic accessory mutation A98G—12.0% was also common. Of the 33 identified PI resistance mutations, 7 were major mutations and 26 minor mutations. The most common major PI-resistance mutations associated with the highest levels of phenotypic resistance were: V82A—7.0% and I54V, M46I, L33I (all 5.0%). The identified accessory PI resistance mutations that either reduce PI susceptibility or increase the replication of viruses containing PI-resistance mutations included: L10I—27.0%, L10V—12.0% and L10F—5.0% (Table 2).

Of the 7 patients with major PI DRMs, high level resistance to Indinavir (IDV), Fosamprenavir (FPV), Lopinavir (LPV) and Nelfinavir (NFV)—to each was found among 4 patients. Intermediate level drug resistance to Saquinavir (SQV) was found among 4 patients, Tipranavir (TPV) and IDV among 3 patients. The HIV among six of the 7 patients with major PI mutations were susceptible to Darunavir with one expressing low level resistance to this drug, and two expressing susceptibility to Tipranavir (Table 3).

**Table 3:** Drug resistance mutations and loss of protease inhibitor (PI) drug options among patients with any major PI mutation

Patient ID	Sex	Age (years)	Viral load (copies/ml)	CD4 (cells/mm^3^)	Duration on ART (years)	Duration on second line ART (years)	HIV-1 sub-type	Identified drug resistance mutations			Level of PI resistance			
								NNRTI	NRTI	PI	High	Intermediate	Low	Susceptible
CoL-1	M	57	339,081	165	9.4	3.6	A		M184V, K65R, A62V	V82S, I54V, M46I, K43T, L24IL, L10F	ATV, FPV, IDV, LPV, NFV	SQV, TPV		DRV
CoL-2	F	58	3886	620	9.3	6.2	A		M184V	V82A, I54V, M46L, L89V, L10F	ATV, FPV, IDV, LPV, NFV	SQV	TPV	DRV
CoL-3	M	38	13,560	130	9.4	6.6	D	A98G, L100I, K103N	M41L, T215Y, L74V	V82A, I54IV, L33F		ATV, FPV, IDV, SQV	LPV, NFV, TPV	DRV
CoL-4	M	34	221,251	130	9.0	6.7	D		M184V, M41L, T215Y	V82A, I54V, M46I, L33F, L24I, L10F	ATV, FPV, IDV, LPV, NFV	SQV	TPV	DRV
CoL-5	M	69	14,127	287	9.2	7.2	A	K103N		V82A		IDV, NFV	ATV, FPV, LPV, SQV	DRV,TPV
CoL-6	F	39	68,537		9.8	7.3	D	K103N, L100I, Y181F	M184L, L74V, M41LM, T215A	V82AV, A71T, L10I, K20T		IDV, LPV, NFV	ATV, FPV, SQV	DRV, TPV
CoL-7	F	40	98,489	97	9.0	7.8	D	G190A, Y181C, K101E	M41L, L210W, K219E, T215C	V82F, I54V, M46I, Q58E, I47V, L10I	IDV, FPV, LPV, NFV	ATV, TPV	DRV, SQV	

*F* Female, *M* Male, *PI* Protease inhibitor, *NRTI* Nucleoside Reverse Transcriptase Inhibitor, *NNRTI* Non-Nucleoside Reverse Transcriptase Inhibitor, *ATV* Atanazavir, *DRV* Darunavir, *FPV* Fosamprenavir, *IDV* Indinavir, *LPV* Lopinavir, *NFV* Nelfinavir, *SQV* Saquinavir, *TPV* Tipranavir

## Conclusions

We found that nearly half of our patients with virological failure had resistance to both NRTIs and NNRTIs, about a fifth had resistance to the three classes of ARVs commonly used in Uganda while a small proportion had no Drug Resistance Mutations (DRMs). NRTIs have fewer side effects and toxicity than NNRTIs, but drug resistance could diminish their efficacy. As expected in Africa, the predominant NRTI mutation observed was M184V [13]. Type 1 TAM M41L causes higher levels of phenotypic and clinical resistance to thymidine analogues and cross resistance to Abacavir, Tenofovir and Didanosine than Type 11 K70R. The M184V and K65R DRMs might be due to the Tenofovir and Lamivudine ART backbone and the triple nucleoside regimen of Abacavir-Zidovudine-Lamivudine that most of our patients were initiated on. Triple nucleoside first-line ART regimens have since been discontinued as they are virologically inferior to a regimen containing Efavirenz (NNRTI) plus two or three NRTIs [14]. The NNRTI mutations observed included K103N, G190A and Y181C which cause high level resistance to Nevirapine and variable resistance to Efavirenz [15].

The observed PI resistance mutations (V82A, V82F, V82S, M46I, M46L and I54V) are clinically significant because they are associated with highest levels of phenotypic resistance and/or strongest evidence for interfering with successful PI therapy. Among the seven patients, these mutations conferred high level resistance to ritonavir boosted Lopinavir and Atazanavir, which are the readily available PIs for second-line ART regimens in our setting. Darunavir, the only susceptible PI tested is still expensive and unavailable in public ART centers in Uganda where PIs are reserved for second-line ART regimens. The emergence of HIV drug resistance is inevitable, owing to the high replication and mutation rates of HIV and ART being a life-long treatment. Therefore, as more people are switched to second-line ART, cases of second-line drug failure will increase and necessitate access to third-line or salvage regimens. In Uganda, routine viral load monitoring is not yet standard care, making early identification of treatment failures to prevent transmission of DRMs impossible. Uganda is yet to recommend HIV genotyping prior to ART initiation, therefore diagnosing transmitted HIV drug resistance is impossible. The strength of our study is the long duration on ART among our participants. A source of selection bias might be that some patients with virological failure might have died before enrolment. We also had limited information on confounders like adherence so our findings may not be generalizable. In conclusion, HIV drug resistance is a major challenge for HIV care programs in resource limited settings that have hitherto concentrated on increasing ART access. Therefore, ART programs in these settings should avail routine HIV viral load monitoring for prompt detection of virological failures, targeted HIV drug resistance testing to detect HIV drug resistance among ART failure as well as access to third-line ARV drugs like Darunavir.

## Abbreviations

HIV: human immunodeficiency virus
ART: antiretroviral therapy
RNA: ribonucleic acid
DRM: drug resistance mutation
WHO: World Health Organisation
IAS: International AIDS Society
NRTI: nucleoside reverse transcriptase inhibitors
NNRTI: non nucleoside reverse transcriptase inhibitors
PI: protease inhibitors
AIDS: acquired immune deficiency syndrome
ARV: antiretroviral
CoLTART: Complications of Long-Term Antiretroviral Therapy
DART: development of antiretroviral therapy in Africa
RIP: recombinant identification program
TDR: transmitted drug resistance
IAS: International AIDS Society
CPR: calibrated population resistance tool
